# High prevalence of integrase mutation L74I in West African HIV-1 subtypes prior to integrase inhibitor treatment

**DOI:** 10.1093/jac/dkaa033

**Published:** 2020-02-27

**Authors:** Kate El Bouzidi, Steven A Kemp, Rawlings P Datir, Fati Murtala-Ibrahim, Ahmad Aliyu, Vivian Kwaghe, Dan Frampton, Sunando Roy, Judith Breuer, Caroline A Sabin, Obinna Ogbanufe, Man E Charurat, David Bonsall, Tanya Golubchik, Christophe Fraser, Patrick Dakum, Nicaise Ndembi, Ravindra K Gupta

**Affiliations:** d1 Division of Infection & Immunity, University College London, London, UK; d2 Institute for Global Health, University College London, London, UK; d3 Department of Medicine, University of Cambridge, Cambridge, UK; d4 Institute of Human Virology Nigeria, Abuja, Nigeria; d5 University of Abuja Teaching Hospital, Abuja, Nigeria; d6 U.S. Centers for Disease Control and Prevention, Diplomatic Drive, Abuja, Nigeria; d7 Institute of Human Virology, University of Maryland School of Medicine, Baltimore, MD, USA; d8 Big Data Institute, University of Oxford, Oxford, UK; d9 Africa Health Research Institute, Durban, KZN, South Africa

## Abstract

**Objectives:**

HIV-1 integrase inhibitors are recommended as first-line therapy by WHO, though efficacy and resistance data for non-B subtypes are limited. Two recent trials have identified the integrase L74I mutation to be associated with integrase inhibitor treatment failure in HIV-1 non-B subtypes. We sought to define the prevalence of integrase resistance mutations, including L74I, in West Africa.

**Methods:**

We studied a Nigerian cohort of recipients prior to and during receipt of second-line PI-based therapy, who were integrase inhibitor-naive. Illumina next-generation sequencing with target enrichment was used on stored plasma samples. Drug resistance was interpreted using the Stanford Resistance Database and the IAS-USA 2019 mutation lists.

**Results:**

Of 115 individuals, 59.1% harboured CRF02_AG HIV-1 and 40.9% harboured subtype G HIV-1. Four participants had major IAS-USA integrase resistance-associated mutations detected at low levels (2%–5% frequency). Two had Q148K minority variants and two had R263K (one of whom also had L74I). L74I was detected in plasma samples at over 2% frequency in 40% (46/115). Twelve (26.1%) had low-level minority variants of between 2% and 20% of the viral population sampled. The remaining 34 (73.9%) had L74I present at >20% frequency. L74I was more common among those with subtype G infection (55.3%, 26/47) than those with CRF02_AG infection (29.4%, 20/68) (*P = *0.005).

**Conclusions:**

HIV-1 subtypes circulating in West Africa appear to have very low prevalence of major integrase mutations, but significant prevalence of L74I. A combination of *in vitro* and clinical studies is warranted to understand the potential implications.

## Introduction

Drug resistance is common amongst individuals with virological failure (VF) of first-line NNRTI-based ART regimens under conditions of infrequent viral load monitoring.^1^^,^^2^ Second-generation integrase inhibitors such as dolutegravir are now recommended for first-line HIV treatment regimens,^3^ following an increase in pre-treatment drug resistance to NNRTI-based regimens globally, including Nigeria.^4–6^ A number of studies have shown that pre-existing integrase resistance,^27^ as assessed using standard lists of mutations derived largely from subtype B data, is rare across globally dominant subtypes.^7^^,^^8^

As dolutegravir-based ART is rolled out globally, a wider range of HIV-1 subtypes will be exposed and the effects of integrase polymorphisms and subtype diversity on the clinical efficacy of these agents are currently not well understood. Although polymorphisms are generally thought to have little impact on viral phenotype, this is not always true, particularly when comparing B with non-B subtypes.^9^ A good example is G118R in integrase, a polymorphism that confers significant integrase strand transfer inhibitor (INSTI) resistance.^10^

Two recent Phase 3 trials of the long-acting injectable second-generation integrase inhibitor cabotegravir and the injectable second-generation NNRTI rilpivirine, FLAIR in ART-naive participants and ATLAS in ART-experienced participants,^11^ found non-inferiority of long-acting injectables compared with oral therapy. However, three participants treated with the long-acting injectable drug experienced VF. All three were infected with HIV-1 subtype A1 and were from Russia. All three had L74I in integrase at both baseline and at VF. At VF the major integrase mutation Q148R occurred in two and G140R in one.^11^

L74 is in the catalytic core domain, which carries out the integrase strand transfer reaction. It is part of a hydrophobic cluster of residues that includes resistance-associated mutations T97 and F121 near the active site.^12^ In the Stanford Resistance Database (https://hivdb.stanford.edu)^13^ L74I is reported to be observed in 3%–20%, depending on subtype. The L74M variant has been included as a minor mutation for the first-generation INSTI raltegravir in the IAS-USA drug resistance mutations list (https://www.iasusa.org/wp-content/uploads/2019/09/27-3-111.pdf), but the L74I variant is not recognized as a resistance-associated mutation.^14^ The Stanford Resistance Database includes L74I in combination with other integrase mutations.^13^ L74I and L74M are assessed together and combined prevalences are often reported^7^ as they have both been shown to enhance integrase inhibitor resistance when present with major INSTI mutations. A recent report suggested that methionine at residue 74 was in closer proximity to T97 and F121 as compared with leucine at position 74 in a modelled subtype C integrase and, of note, L74F was found to contribute to high-level dolutegravir resistance when combined with major mutations G140S and Q148H.^15^

We studied a Nigerian cohort of people living with HIV in whom the West African CRF02_AG and G subtypes account for the majority of infections.^16^ We aimed to determine the prevalence of INSTI resistance, as well as the prevalence and dynamics of L74I, in this setting.

## Patients and methods

Study participants were selected from an HIV-positive second-line treatment cohort at the University of Abuja Teaching Hospital (UATH) in Abuja, Nigeria. Informed consent was obtained from all participants and ethics approval for virological testing was obtained from the Nigeria National Research Ethics Committee of Nigeria (NHREC/01/01/2007). Ethics approval was also obtained from the ethics board of UCL, UK. The Institute of Human Virology Nigeria (IHVN) database was used to identify people living with HIV (PLWH) aged >15 years who had attended UATH and received a first-line ART regimen of two NRTIs and one NNRTI, followed by a second-line ART regimen of two NRTIs and one PI (lopinavir or atazanavir). Participants were included in the study if they: (i) had experienced first-line VF, defined as HIV RNA >1000 copies/mL at least 6 months after first-line ART initiation; (ii) had a stored plasma sample that was obtained during first-line VF, prior to switching to a second-line regimen; and (iii) had a whole HIV genome sequence successfully generated from the first-line VF sample. If participants had subsequent stored plasma samples from first-line or second-line VF these were also included where possible. CD4 cell count and HIV-1 RNA quantitation were performed at the IHVN laboratories. For next-generation sequencing, manual nucleic acid extraction was done at University College London (UCL) using the QIAamp Viral RNA Mini Kit, (QIAGEN, Hilden, Germany) with a plasma input volume of 0.5–1.5 mL. The first strand of cDNA was synthesized using SuperScript IV reverse transcriptase (Invitrogen, Waltham, MA, USA), followed by NEBNext second-strand cDNA synthesis (E6111, New England Biolabs GmbH, Frankfurt, Germany). Sample libraries were prepared as per the SureSelect^XT^ automated target enrichment protocol (Agilent Technologies, Santa Clara, CA, USA) with in-house HIV baits. Whole-genome deep sequencing was performed using the Illumina Miseq platform (San Diego, CA, USA).

Poor-quality reads were identified and removed using TrimGalore v0.6.4.^17^ A set of 170 HIV-1 subtypes/circulating recombinant forms (CRFs) were downloaded from the Los Alamos database (https://www.hiv.lanl.gov) and the trimmed reads were compared with this database using BLAST to identify the closest reference.^18^ Trimmed reads were mapped to the closest reference genome using the Burrows–Wheeler aligner.^19^ Duplicate reads were removed from the BAM files with Picard (http://broadinstitute.github.io/picard) and a consensus sequence of nucleic acids with a minimum whole-genome coverage of 20× was generated with BCFtools using a 50% threshold.^20^ Consensus sequences were used to determine the codon usage at integrase position 74 and subtype was assigned using REGA v3.0.^21^ The presence and frequency of IAS-USA resistance-associated mutations in integrase, in addition to L74I, was assessed. Mutations were included if they were present at over 2% frequency within the read mixture at that position and present on at least two reads. A threshold of 2% is supported by a study evaluating different analysis pipelines, which reported fewer discordances over this cut-off.^22^ In addition, inspection of the mean read depth across regions of interest in the present study showed that a cut-off of <2% would not include sufficient reads to provide accurate assumptions regarding resistance mutations. An in-house custom script was used to identify SNPs at each position by BLAST analysis of individual HIV *pol* against the HXB2 reference genome. SNPs were identified and then translated to codons across all regions. Statistical analysis was performed in Stata v13.1 (StataCorp LLC, College Station, TX, USA) and SAS v9.4 (SAS Institute, Cary, NC, USA).

## Results

Overall, 115 participants had a total of 163 plasma samples that yielded HIV *pol* sequences. Two participants had a sample obtained prior to receiving any ART, 72 participants had samples from first-line ART only (two had multiple first-line samples), 14 participants had samples from second-line ART only (four had multiple second-line samples) and 27 participants had samples obtained during first-line and second-line ART (Table S1, available as Supplementary data at *JAC* Online). The median sequencing coverage of all samples at the whole-genome level was 583 reads per base (IQR = 136–1313), 595 (IQR = 125–1324) for the *pol* gene and 735 (IQR = 162–1593) for the integrase L74 codon. Detailed coverage data are shown in Table S2. Four participants had major IAS-USA integrase resistance-associated mutations detected at low levels (2%–5% frequency). Two had Q148K and two had R263K minority variants (one of whom also had L74I). Another six participants had minor IAS-USA integrase resistance-associated mutations. Four had T97A [this was the consensus in three of them and a minority variant in one (who also had L74I)], another participant had an E138K minority variant and another had a G140A minority variant. The most common integrase polymorphism was E157Q, which was detected in 12 participants (6 of these also had L74I).

Forty-six (40%) of the participants had L74I detected at >2% frequency in at least one plasma sample. The participant characteristics of those with and without an L74I mutation were similar, with an overall median age of 30 years, two-thirds were female and the median CD4 cell count at first-line VF was 142 cells/mm^3^ (IQR = 64–246) (see Table 1).

**Table 1.** Participant characteristics at first-line ART failure

	All, *N = *115	L74I present at >2% frequency, *N = *46	L74I not detected, *N = *69	*P* ^a^
Age (years), median (IQR)	30 (26–39)	32 (27–40)	30 (26–37)	0.28
<30, *n* (%)	48 (41.7)	14 (30.4)	34 (49.3)	0.05
>30, *n* (%)	67 (58.2)	32 (69.6)	35 (50.7)	
Sex, *n* (%)				
female	78 (67.8)	33 (71.7)	45 (65.2)	0.46
male	37 (32.2)	13 (28.3)	24 (34.8)	
CD4 cell count (cells/mm^3^), median (IQR)^b^	142 (64–246)	153 (112–256)	133 (47–214)	0.06
<200, *n* (%)	77 (67.5)	28 (60.9)	49 (72.1)	0.21
>200, *n* (%)	37 (32.5)	18 (39.1)	19 (27.9)	
HIV-1 RNA (log_10_ copies/mL), median (IQR)^b^	4.94 (4.36–5.37)	4.83 (4.40–5.19)	5.00 (4.35–5.48)	0.09
<100 000, *n* (%)	65 (57.5)	31 (68.9)	34 (50.0)	0.05
>100 000, *n* (%)	48 (42.5)	14 (31.1)	34 (50.0)	
Time on ART (months), median (IQR)	32.9 (19.0–49.6)	34.7 (19.0–46.2)	31.0 (18.8–50.5)	0.74
<1 year, *n* (%)	16 (13.9)	5 (10.9)	11 (15.9)	0.74
1–2 years, *n* (%)	25 (21.7)	11 (23.9)	14 (20.3)	
2–3 years, *n* (%)	24 (20.9)	9 (19.6)	15 (21.7)	
3–4 years, *n* (%)	20 (17.4)	10 (21.7)	10 (14.5)	
>4 years, *n* (%)	30 (26.1)	11 (23.9)	19 (27.5)	
HIV-1 subtype, *n* (%)				
CRF02_AG	68 (59.1)	20 (43.5)	48 (69.6)	0.005
G	47 (40.9)	26 (56.5)	21 (30.4)	

a *χ* ^2^ or Mann–Whitney test, as appropriate.

b Missing data: CD4 cell count, 1 (L74I, 0; no L74I, 1); and HIV-1 RNA, 2 (L74I, 1; no L74I, 1).

Considering the first timepoint at which L74I was detected for each participant, the median frequency of L74I was 90.6% (IQR = 17.8–98.7). Twelve of the 46 participants (26.1%) had low-level minority variants of between 2% and 20% of the viral population sampled (seven with 2%–5% frequency, four with 5%–10% and one with 10%–20%). The remaining 73.9% (34/46) had L74I present at >20% frequency (the usual Sanger sequencing threshold of detection). This comprised 4 participants with 20%–50% frequency, 6 with 50%–90% frequency and 24 in whom L74I was fixed at >90% frequency at that position. There was a subtype difference in L74I prevalence, with 55.3% (26/47) of participants with subtype G infection having the L74I mutation detected in at least one plasma sample, compared with 29.4% (20/68) of those with CRF02_AG infection (*P = *0.005). There was a similar frequency of L74I among the subtypes, with a median frequency of 93.0% (IQR = 26.9–99.0) in CRF02_AG and 89.2% (IQR = 8.1–98.2) (*P = *0.62) in subtype G.

Thirty-three participants had more than one plasma sample and therefore intra-host changes could be evaluated. Eight participants had L74I as the majority allele (>85% frequency) in every sample (Table 2). Four had low-level minority variants of less than 10% frequency and in two of these L74I was not detected in the preceding plasma samples. Two participants had reversion to low (<2%) or no L74I in subsequent samples.

**Table 2.** Participants with L74I and longitudinal sampling

Participant	Subtype	Sample	ART line	Viral load (copies/mL)	*pol* drug resistance mutations (consensus sequence)	Integrase L74I (%)	Integrase L74I evolution
1	G	1	first	373 000	RT: M184V, K103N, Y318F	99.2	fixed majority
		2	first	274 500	RT: K70R, M184V, K219Q, K103N, Y318F	88.4	
2	G	1	first	532 000	RT: D67N, K70R, M184V, T215I, K219E, Y181C	99.6	fixed majority
		2	second	36 000	RT: M184V, T215F, K219E, V179VLM, Y181C	90.9	
3	G	1	first	29 000	RT: K70R, M184V, A98G, V108I, Y181C	95.6	fixed majority
		2	second	4300	RT: K70R, M184V, T215F, K219Q, V108I, Y181C, N348I	93.3	
4	G	1	first	25 000	RT: M184V, K103N, K238T	99.5	fixed majority
		2	second	1 450 000	none	99.8	
5	G	1	first	370 000	RT: M184V, K103N, Y318F	99.2	fixed majority
		2	second	275 000	RT: K70R, M184V, K219Q, K103N, Y318F	88.4	
6	G	1	first	28 000	RT: M41L, D67N, T69D, K70R, V75M, M184V, T215F, K219Q, A98G, K103N, V179E, K238T, Y318F	85.5	fixed majority
		2	first	63 000	RT: M41L, D67N, T69D, K70R, V75M, M184V, T215F, K219Q, A98G, K103N, V179E, K238T, Y318F	99.1	
		3	first	442 000	RT: M41L, D67N, T69D, K70R, V75M, M184V, T215FL, K219Q, A98G, K103N, V179E, K238T, Y318F	98.9	
		4	second	5800	RT: M41L, D67N, T69D, V75M, M184V, T215IV, K219Q, A98G, K103N, V179E, K238T, Y318F	97.7	
7	AG	1	first	48 000	RT: M184V, K103N, P225H	98.1	fixed majority
		2	second	11 000	RT: M184V, K103N	94.7	
8	AG	1	first	241 000	RT: K103N, H221Y	99.5	fixed majority
		2	first	250 000	RT: Y181C, H221Y	99.0	
		3	second	4400	RT: K103N, Y181C, H221Y	92.3	
9	G	1	first	272 000	RT: K70E, Y115F, M184V, Y181C, G190A, H221Y	90.0	revertant majority
		2	second	24 000	RT: K70E, Y115F, M184V, Y181C, G190A, H221YPro: M46I, I54V, V82S, L24I, L33F	ND	
10	G	1	first	59 000	RT: K65R, M184V, K219E, Y181C, H221Y	2.2	fixed minority
		2	second	1700	RT: K65R, M184V, K219E, Y181C, H221YPro: I54V, V82A, L10F	2.7	
11	G	1	second	5700	RT: M41L, V75I, M184V, T215F, K101H, G190A, N348I	3.6	fixed minority
		2	second	384 000	RT: M41L, D67N, V75I, M184V, T215F, G190A, N348I	2.1	
12	AG	1	second	5700	RT: M41L, V75I, M184V, T215F, K101H, G190A, N348I	3.6	fixed minority
		2	second	384 000	RT: M41L, D67N, V75I, T215F, G190A, N348I	2.1	
13	G	1	first	20 000	RT: M184V, K101E, G190A	ND	emergent minority
		2	second	118 000	none	8.1	
14	G	1	first	77 000	RT: M41L, E44D, D67N, K70R, L74V, M184V, T215Y, Y181C, G190A, N348IPro: L90M	39.2	revertant minority
		2	second	56 000	RT: M41L, E44D, D67N, T69D, K70R, L74V, M184V, T215Y, Y181C, G190A, N348IPro: M46I, 154V, V82A, L90M	2.4	

RT, reverse transcriptase; Pro, protease; AG, CRF02_AG; ND, not detected.

Codon usage at the integrase 74 position was examined using consensus sequences. All sequences with L74I at consensus (27%, 31/115) had the trinucleotide ATA at this position. The remaining consensus sequences were all L74L. The trinucleotides CTG or TTG were the consensus codon in 60% (69/115), requiring two nucleotide changes to result in an amino acid substitution from leucine to isoleucine, and 13% (15/115) had either TTA or CTA, requiring only one nucleotide change to mutate to L74I. Synonymous changes in codon usage were noted in two of the participants from whom more than one sample was available. One changed from TTA to CTA (both requiring one further nucleotide substitution to code for I) and the other from CTG to CTA (from requiring two nucleotide substitutions to code for I to just one). There was no significant subtype difference in the number of substitutions required to mutate from L74L to L74I (*P = *0.13).

## Discussion

As we enter an era when dolutegravir-based ART will be used for tens of millions of PLWH, it is vital to understand determinants of treatment failure and drug resistance. To date, information on integrase sequences by next-generation sequencing in subtypes dominating in West Africa has been extremely limited. Recently, a series of VF patients in a trial of the integrase inhibitor cabotegravir were found to harbour L74I (a polymorphic mutation that is weakly selected for by INSTI therapy) in addition to other major integrase inhibitor drug resistance mutations. This prompted us to evaluate not only major integrase inhibitor mutations, but also L74I.

Reassuringly, we found that major resistance mutations to integrase inhibitors were very rare in this study population that had extensive NRTI and NNRTI resistance following VF with NNRTI-based regimens. One individual out of 115 had both L74I and the signature dolutegravir mutation R263K detected by next-generation sequencing,^23–26^ though R263K was a minority variant.

We found over a third of the integrase inhibitor-naive HIV-positive participants in our study had the integrase L74I mutation and that L74I was more common in HIV-1 subtype G than CRF02_AG. This is the first time that such a high prevalence of L74I mutations has been reported in West African G and AG subtypes, with potential implications for the effectiveness of dolutegravir, which is now being rolled out as part of the first-line treatment in this setting, where there is less frequent viral load monitoring and less access to genotypic resistance testing.

Uniquely, in this study we were able to assess the frequency of viral variants with L74I in longitudinal samples from multiple individuals. In most cases there was no change in variant frequencies, consistent with L74I being transmitted between individuals following a founder effect and L74I reverting rarely, even during second-line ART failure. This would be consistent with a lack of fitness cost of L74I in the absence of drug pressure.

A limitation of this study was that our patient group were mainly ART-experienced and as such there may be a different prevalence of L74I in treatment-naive individuals. Furthermore, although L74I was associated with VF in two studies including the long-acting injectable cabotegravir, it is not known whether L74I contributed to VF or what the impact on dolutegravir might be.

*In vitro* studies are needed to determine whether L74I facilitates high-level INSTI resistance in non-B subtypes and clinical studies are necessary to determine whether L74I at baseline impacts clinical or virological outcomes on integrase inhibitors, even when short-term outcomes in cross-sectional studies appear favourable.

## Funding

K.E.B. is supported by Wellcome Trust award number 170461. N.N. is supported by NIH R01 AI147331-01. R.K.G. is supported by a Wellcome Trust Senior Fellowship in Clinical Science (WT108082AIA). This study was supported by the President’s Emergency Plan for AIDS Relief (PEPFAR) through the Centers for Disease Control and Prevention (CDC) under the terms of U2G GH002099-01 and PA GH17-1753 (ACHIEVE).

## Transparency declarations

C.A.S. has received honoraria from Gilead Sciences, ViiV Healthcare and Janssen-Cilag for membership of Data Safety and Monitoring Boards and Advisory Boards, and for preparation of educational materials. R.K.G. has received speaker fees for *ad hoc* consulting from Gilead and ViiV Healthcare. All other authors: none to declare.

## Supplementary data

Tables S1 and S2 are available as Supplementary data at JAC Online.

## Supplementary Material

dkaa033_Supplementary_Data

## References

[1] Gregson J, Kaleebu P, Marconi VC et al Occult HIV-1 drug resistance to thymidine analogues following failure of first-line tenofovir combined with a cytosine analogue and nevirapine or efavirenz in sub Saharan Africa: a retrospective multi-centre cohort study. Lancet Infect Dis 2017; 17: 296–304. 27914856 10.1016/S1473-3099(16)30469-8 PMC5421555

[2] TenoRes Study Group. Global epidemiology of drug resistance after failure of WHO recommended first-line regimens for adult HIV-1 infection: a multicentre retrospective cohort study. Lancet Infect Dis 2016; 16: 565–75. 26831472 10.1016/S1473-3099(15)00536-8 PMC4835583

[3] WHO. *Guidelines on the Public Health Response to Pretreatment HIV Drug Resistance* 2017 http://who.int/hiv/pub/guidelines/hivdr-guidelines-2017/.

[4] Gupta RK, Jordan MR, Sultan BJ et al Global trends in antiretroviral resistance in treatment-naive individuals with HIV after rollout of antiretroviral treatment in resource-limited settings: a global collaborative study and meta-regression analysis. Lancet 2012; 380: 1250–8. 22828485 10.1016/S0140-6736(12)61038-1 PMC3790969

[5] Gupta RK, Gregson J, Parkin N et al HIV-1 drug resistance before initiation or re-initiation of first-line antiretroviral therapy in low-income and middle-income countries: a systematic review and meta-regression analysis. Lancet Infect Dis 2018; 18: 346–55. 29198909 10.1016/S1473-3099(17)30702-8 PMC5835664

[6] Chaplin B, Akanmu AS, Inzaule SC et al Association between HIV-1 subtype and drug resistance in Nigerian infants. J Antimicrob Chemother 2019; 74: 172–6. 30260417 10.1093/jac/dky380

[7] Inzaule SC, Hamers RL, Noguera-Julian M et al Primary resistance to integrase strand transfer inhibitors in patients infected with diverse HIV-1 subtypes in sub-Saharan Africa. J Antimicrob Chemother 2018; 73: 1167–72. 29462322 10.1093/jac/dky005

[8] Derache A, Iwuji CC, Danaviah S et al Predicted antiviral activity of tenofovir versus abacavir in combination with a cytosine analogue and the integrase inhibitor dolutegravir in HIV-1-infected South African patients initiating or failing first-line ART. J Antimicrob Chemother 2019; 74: 473–9. 30380053 10.1093/jac/dky428 PMC6337894

[9] Han YS, Mesplede T, Wainberg MA. Differences among HIV-1 subtypes in drug resistance against integrase inhibitors. Infect Genet Evol 2016; 46: 286–91. 27353185 10.1016/j.meegid.2016.06.047

[10] Quashie PK, Oliviera M, Veres T et al Differential effects of the G118R, H51Y, and E138K resistance substitutions in different subtypes of HIV integrase. J Virol 2015; 89: 3163–75. 25552724 10.1128/JVI.03353-14 PMC4337543

[11] Fernandez C, van Halsema CL. Evaluating cabotegravir/rilpivirine long-acting, injectable in the treatment of HIV infection: emerging data and therapeutic potential. HIV AIDS *(Auckl)* 2019; 11: 179–92. 10.2147/HIV.S184642 PMC6682757 31447590

[12] Rogers L, Obasa AE, Jacobs GB et al Structural implications of genotypic variations in HIV-1 integrase from diverse subtypes. Front Microbiol 2018; 9: 1754. 30116231 10.3389/fmicb.2018.01754 PMC6083056

[13] Liu TF, Shafer RW. Web resources for HIV type 1 genotypic-resistance test interpretation. Clinical Infect Dis 2006; 42: 1608–18. 16652319 10.1086/503914 PMC2547473

[14] Wensing AM, Calvez V, Ceccherini-Silberstein F et al 2019 update of the drug resistance mutations in HIV-1. Top Antivir Med 2019; 27: 111–21. 31634862 PMC6892618

[15] Hachiya A, Kirby KA, Ido Y et al Impact of HIV-1 integrase L74F and V75I mutations in a clinical isolate on resistance to second-generation integrase strand transfer inhibitors. Antimicrob Agents Chemother 2017; 61: e00315-17. 28533248 10.1128/AAC.00315-17 PMC5527620

[16] Lihana RW, Ssemwanga D, Abimiku A et al Update on HIV-1 diversity in Africa: a decade in review. AIDS Rev 2012; 14: 83–100. 22627605

[17] Martin M. Cutadapt removes adapter sequences from high-throughput sequencing reads. EMBnet J 2011; 17: 10–2.

[18] Gish W, States DJ. Identification of protein coding regions by database similarity search. Nat Genet 1993; 3: 266–72. 8485583 10.1038/ng0393-266

[19] Li H, Durbin R. Fast and accurate short read alignment with Burrows-Wheeler transform. Bioinformatics 2009; 25: 1754–60. 19451168 10.1093/bioinformatics/btp324 PMC2705234

[20] Li H. A statistical framework for SNP calling, mutation discovery, association mapping and population genetical parameter estimation from sequencing data. Bioinformatics 2011; 27: 2987–93. 21903627 10.1093/bioinformatics/btr509 PMC3198575

[21] Pineda-Pena AC, Faria NR, Imbrechts S et al Automated subtyping of HIV-1 genetic sequences for clinical and surveillance purposes: performance evaluation of the new REGA version 3 and seven other tools. Infect Genet Evol 2013; 19: 337–48. 23660484 10.1016/j.meegid.2013.04.032

[22] Perrier M, Desire N, Storto A et al Evaluation of different analysis pipelines for the detection of HIV-1 minority resistant variants. PLoS One 2018; 13: e0198334. 29856864 10.1371/journal.pone.0198334 PMC5983569

[23] Ahmed N, Flavell S, Ferns B et al Development of the R263K mutation to dolutegravir in an HIV-1 subtype D virus harboring 3 class-drug resistance. Open Forum Infect Dis 2019; 6: ofy329. 30648124 10.1093/ofid/ofy329 PMC6329901

[24] Quashie PK, Mesplede T, Han YS et al Characterization of the R263K mutation in HIV-1 integrase that confers low-level resistance to the second-generation integrase strand transfer inhibitor dolutegravir. J Virol 2012; 86: 2696–705. 22205735 10.1128/JVI.06591-11 PMC3302270

[25] Lepik KJ, Harrigan PR, Yip B et al Emergent drug resistance with integrase strand transfer inhibitor-based regimens. AIDS 2017; 31: 1425–34. 28375875 10.1097/QAD.0000000000001494

[26] Blanco JL, Marcelin AG, Katlama C et al Dolutegravir resistance mutations: lessons from monotherapy studies. Curr Opin Infect Dis 2018; 31: 237–45. 29634660 10.1097/QCO.0000000000000453

[27] Collier D, Monit C, Gupta RK The impact of HIV-1 drug resistance on the global treatment landscape. Cell Host Microbe 2019; 26: 48–60. 31295424 10.1016/j.chom.2019.06.010

