# Supplementary material for: High prevalence of integrase mutation L74I in West African HIV-1 subtypes prior to integrase inhibitor treatment
Source: J Antimicrob Chemother. 2020 Feb 27;75(6):1575–9. doi: 10.1093/jac/dkaa033 (PMC7225870; doi:10.1093/jac/dkaa033)
Supplement: dkaa033_Supplementary_Data [file dkaa033_supplementary_data.docx]

**Supplementary data**

**Table S1.** **Number of samples per participant from each timepoint**

| Number of samples from each timepoint | Number of participants | Number of samples |
| --- | --- | --- |
| 1 x ART-naïve sample | 2 | 2 |
| 1 x first-line sample | 71 | 71 |
| 2 x first-line samples* | 1 | 2 |
| 3 x first-line samples* | 1 | 3 |
| 1 x second-line sample | 9 | 9 |
| 2 x second-line samples* | 3 | 6 |
| 3 x second-line samples* | 1 | 3 |
| 1 x first-line sample + 1 x second-line sample* | 19 | 38 |
| 1 x first-line sample + 2 x second-line samples* | 4 | 12 |
| 1 x first-line sample + 5 x second-line samples* | 1 | 6 |
| 2 x first-line samples + 1 x second-line sample* | 1 | 3 |
| 2 x first-line samples + 2 x second-line samples* | 1 | 4 |
| 3 x first-line samples + 1 x second-line sample* | 1 | 4 |
| **Total** | **115** | **163** |

*longitudinal participants with more than one sample

**Table S2. Sequencing coverage of all samples**

| Sample number | Subtype | Whole genome  (mean read depth) | Pol gene  (mean read depth) | Integrase L74 codon  (mean read depth) |
| --- | --- | --- | --- | --- |
| 1 | AG | 53 | 47 | 62 |
| 4 | AG | 183 | 171 | 312 |
| 5 | AG | 76 | 58 | 57 |
| 6 | AG | 55 | 50 | 75 |
| 8 | AG | 424 | 416 | 505 |
| 9 | AG | 154 | 155 | 161 |
| 11 | G | 218 | 200 | 328 |
| 12 | AG | 233 | 212 | 291 |
| 13 | AG | 497 | 468 | 623 |
| 14 | G | 359 | 345 | 452 |
| 16 | AG | 793 | 750 | 906 |
| 17 | G | 1491 | 1465 | 1766 |
| 18 | AG | 262 | 236 | 262 |
| 19 | AG | 1253 | 1193 | 1562 |
| 20 | G | 366 | 293 | 335 |
| 21 | AG | 561 | 537 | 795 |
| 22 | G | 558 | 491 | 545 |
| 24 | AG | 1127 | 1172 | 1496 |
| 25 | AG | 628 | 595 | 705 |
| 26 | AG | 78 | 63 | 66 |
| 28 | G | 124 | 84 | 76 |
| 29 | AG | 4609 | 4132 | 5947 |
| 30 | AG | 139 | 121 | 175 |
| 31 | AG | 32 | 37 | 34 |
| 32 | AG | 1759 | 1697 | 2081 |
| 33 | AG | 913 | 1019 | 1271 |
| 34 | G | 36 | 30 | 38 |
| 35 | AG | 1318 | 1524 | 2241 |
| 36 | AG | 389 | 327 | 445 |
| 37 | AG | 1605 | 1645 | 2094 |
| 38 | AG | 2311 | 2230 | 3201 |
| 39 | AG | 862 | 834 | 983 |
| 40 | AG | 2978 | 3120 | 4087 |
| 41 | AG | 2988 | 2893 | 3403 |
| 42 | AG | 3787 | 3639 | 4897 |
| 43 | G | 174 | 163 | 232 |
| 44 | AG | 1135 | 1131 | 1618 |
| 47 | AG | 893 | 895 | 987 |
| 48 | AG | 4803 | 4939 | 7678 |
| 49 | G | 85 | 68 | 138 |
| 54 | AG | 624 | 617 | 639 |
| 58 | G | 121 | 80 | 68 |
| 60 | AG | 354 | 352 | 426 |
| 61 | G | 89 | 50 | 47 |
| 62 | AG | 1751 | 1820 | 2202 |
| 63 | AG | 947 | 1004 | 1250 |
| 64 | AG | 57 | 44 | 56 |
| 65 | AG | 891 | 815 | 983 |
| 66 | AG | 31 | 27 | 30 |
| 67 | G | 37 | 36 | 38 |
| 68 | AG | 1363 | 1350 | 1781 |
| 69 | AG | 31 | 27 | 55 |
| 70 | AG | 627 | 712 | 793 |
| 71 | AG | 583 | 611 | 649 |
| 72 | AG | 552 | 555 | 758 |
| 73 | G | 78 | 66 | 67 |
| 74 | AG | 1425 | 1436 | 1982 |
| 75 | AG | 191 | 219 | 221 |
| 76 | G | 89 | 69 | 67 |
| 77 | AG | 77 | 72 | 62 |
| 78 | AG | 516 | 522 | 889 |
| 79 | AG | 908 | 915 | 1103 |
| 80 | AG | 218 | 200 | 358 |
| 81 | AG | 718 | 659 | 725 |
| 82 | AG | 54 | 39 | 61 |
| 83 | AG | 89 | 69 | 206 |
| 84 | AG | 135 | 147 | 172 |
| 85 | G | 1916 | 1778 | 2401 |
| 86 | AG | 46 | 51 | 59 |
| 87 | AG | 161 | 129 | 128 |
| 88 | AG | 905 | 987 | 1251 |
| 90 | AG | 55 | 55 | 49 |
| 91 | G | 224 | 215 | 131 |
| 92 | G | 1366 | 1318 | 1473 |
| 93 | G | 84 | 57 | 74 |
| 97 | AG | 75 | 80 | 60 |
| 98 | G | 56 | 52 | 57 |
| 99 | AG | 909 | 936 | 1100 |
| 100 | AG | 1234 | 1222 | 1470 |
| 101 | G | 989 | 929 | 1201 |
| 108 | AG | 94 | 99 | 105 |
| 109 | G | 94 | 92 | 128 |
| 111 | G | 63 | 61 | 77 |
| 114 | AG | 136 | 153 | 199 |
| 115 | AG | 64 | 66 | 49 |
| 116 | AG | 65 | 68 | 100 |
| 119 | AG | 396 | 362 | 507 |
| 120 | AG | 89 | 92 | 126 |
| 121 | AG | 824 | 939 | 1151 |
| 122 | G | 384 | 375 | 443 |
| 123 | G | 254 | 219 | 309 |
| 124 | G | 241 | 217 | 233 |
| 125 | G | 40 | 35 | 52 |
| 126 | G | 312 | 303 | 335 |
| 127 | G | 186 | 174 | 198 |
| 128 | G | 371 | 353 | 414 |
| 129 | AG | 604 | 684 | 899 |
| 133 | AG | 157 | 178 | 237 |
| 134 | AG | 41 | 36 | 45 |
| 136 | AG | 36 | 35 | 38 |
| 141 | G | 242 | 238 | 300 |
| 143 | AG | 336 | 337 | 364 |
| 144 | AG | 553 | 513 | 703 |
| 145 | AG | 1145 | 1237 | 1418 |
| 147 | AG | 612 | 671 | 919 |
| 148 | AG | 1441 | 1524 | 2428 |
| 149 | AG | 737 | 731 | 737 |
| 150 | G | 757 | 746 | 893 |
| 151 | G | 621 | 608 | 686 |
| 152 | G | 530 | 488 | 560 |
| 153 | G | 871 | 837 | 1035 |
| 154 | AG | 570 | 537 | 771 |
| 155 | G | 1280 | 1259 | 1279 |
| 156 | AG | 26 | 28 | 33 |
| 157 | AG | 860 | 959 | 1264 |
| 158 | G | 30 | 29 | 24 |
| 159 | AG | 1228 | 1378 | 1579 |
| 160 | AG | 94 | 99 | 163 |
| 161 | G | 89 | 87 | 103 |
| 162 | G | 337 | 329 | 410 |
| 163 | AG | 580 | 577 | 764 |
| 165 | AG | 89 | 78 | 101 |
| 166 | AG | 1148 | 1229 | 1608 |
| 168 | AG | 1262 | 1281 | 1456 |
| 169 | AG | 1423 | 1543 | 1971 |
| 170 | AG | 1322 | 1109 | 1489 |
| 171 | AG | 2597 | 2632 | 4107 |
| 172 | AG | 1308 | 1338 | 2004 |
| 173 | AG | 1820 | 1980 | 2508 |
| 175 | AG | 2682 | 2909 | 3482 |
| 176 | G | 1870 | 1871 | 2401 |
| 177 | AG | 2593 | 2744 | 3281 |
| 178 | AG | 1743 | 1641 | 2164 |
| 179 | G | 1973 | 1910 | 2221 |
| 181 | AG | 1321 | 1331 | 1366 |
| 182 | G | 1072 | 1078 | 1381 |
| 183 | AG | 715 | 786 | 1163 |
| 184 | AG | 1107 | 1312 | 1980 |
| 185 | G | 497 | 511 | 695 |
| 186 | AG | 4906 | 4874 | 7936 |
| 187 | AG | 988 | 1002 | 1292 |
| 188 | AG | 855 | 843 | 844 |
| 189 | AG | 1690 | 1802 | 2452 |
| 190 | AG | 2288 | 2419 | 3428 |
| 191 | G | 1828 | 1956 | 2035 |
| 193 | AG | 3910 | 3939 | 6882 |
| 194 | AG | 1350 | 1380 | 1448 |
| 196 | G | 2638 | 2724 | 4037 |
| 199 | G | 1357 | 1249 | 1371 |
| 200 | AG | 1287 | 1475 | 1881 |
| 201 | AG | 2132 | 2233 | 3481 |
| 202 | AG | 66 | 65 | 93 |
| 203 | AG | 36 | 28 | 22 |
| 204 | AG | 1937 | 1810 | 1917 |
| 205 | AG | 451 | 411 | 395 |
| 206 | AG | 498 | 520 | 625 |
| 207 | AG | 2105 | 2164 | 3192 |
| 208 | AG | 1832 | 1861 | 2673 |
| 209 | AG | 995 | 995 | 1229 |
| 210 | AG | 749 | 703 | 735 |
| 211 | AG | 2230 | 2196 | 3272 |
| 212 | G | 1641 | 1563 | 1898 |
| 213 | AG | 2864 | 2920 | 4493 |
